# Quantum-like decreased embryogenesis time with increased cold exposure time

**DOI:** 10.1038/s41598-018-35396-2

**Published:** 2019-02-04

**Authors:** Tarushika Vasanthan, Joshua P. Nederveen, Jonathon Stone

**Affiliations:** 1 Department of Biology, McMaster University, 1280 Main Street West, Hamilton, ON L8S 4K1 Canada; 2 Origins Institute, McMaster University, 1280 Main Street West, Hamilton, ON L8S 4M1 Canada; 3 Department of Kinesiology, McMaster University, 1280 Main Street West, Hamilton, ON L8S 4K1 Canada

## Abstract

Three theoretical models have been proposed to explain lifespan extension resulting from exposure to extreme conditions in microscopic animals: individuals become completely dormant and stop aging, continue to age or age but at a diminished rate. Here we show that the earliest life history stages, embryonic cell divisions, in the tardigrade species *Hypsibius dujardini* are retarded when eggs are reared at 0 °C. Compared to control specimens reared at 22 °C, juveniles that hatched from eggs exposed to 0 °C for 4 days and returned to 22 °C experienced a three-day lag, indicating that their biological age was less than their chronological age. As cold exposure duration increased (days = 10, 20, 40), incubation period at 22 °C decreased incrementally (days = 3, 2, 1), suggesting that tardigrades involve a threshold-determined, quantum-like, energetic-based system for controlling embryogenesis.

## Introduction

An anhydrobiotic state is a condition characterised by reduced, possibly lacking, metabolic activity induced by desiccation at any stage in life history. Anhydrobiosis enables micrometazoans like rotifers, nematodes and tardigrades to tolerate extremely arid conditions that otherwise would be lethal^1^. Each anhydrobiotic individual can remain dormant in a desiccated state for weeks, months or even years, until surrounding conditions become favorable for re-animation^2^. During desiccation onset, cell protectants like trehalose, heat shock proteins and intrinsically disordered proteins are synthesized to assist in molecular and cellular stabilization^3–5^. Successful resurrection following desiccation is favored by gradual dehydration^1,6^.

Desiccation tolerance effects have been documented at the molecular level^7^, but effects on life history have received less attention. Three explanations have been proposed^8^. The ‘Sleeping Beauty’ model states that metabolic and physiological processes halt in desiccated organisms; no aging occurs, in analogy with the famous fairy tale, wherein a princess sleeps for 100 years and awakens at the same point in life history as when she entered her century-long slumber^8,9^. The ‘Rip van Winkle’ model states that metabolic and physiological processes continue in desiccated organisms; aging continues, in analogy with the famous short story, wherein a villager sleeps for 20 years and awakens accordingly advanced in age relative to the point in life history in which he entered his two-decade repose^8,9^. The third model involves the suggestion that metabolic and physiological processes continue at a lower rate in desiccated organisms; aging continues but in a diminished manner^8,9^.

The Sleeping Beauty model has been supported by studies on two rotifer species (*Macrotrachela quadricornifera* and *Adineta ricciae*) and one tardigrade species (*Milnesium tardigradum*), wherein desiccated then rehydrated specimens lived for as many active days as did control specimens^9–11^. The Rip van Winkle and partial aging models have been supported by studies on one nematode species (*Panagrolaimus rigidus*)^8,12^. Effects from desiccation on lifespan thus have been documented with adult micrometazoans, but whether cold temperatures could produce similar effects at earlier life history stages remains unknown. We therefore used the tardigrade species *Hypsibius dujardini* to test whether embryonic cell divisions are retarded when eggs are reared at 0 °C.

## Results and Discussion

**Figure 1:**
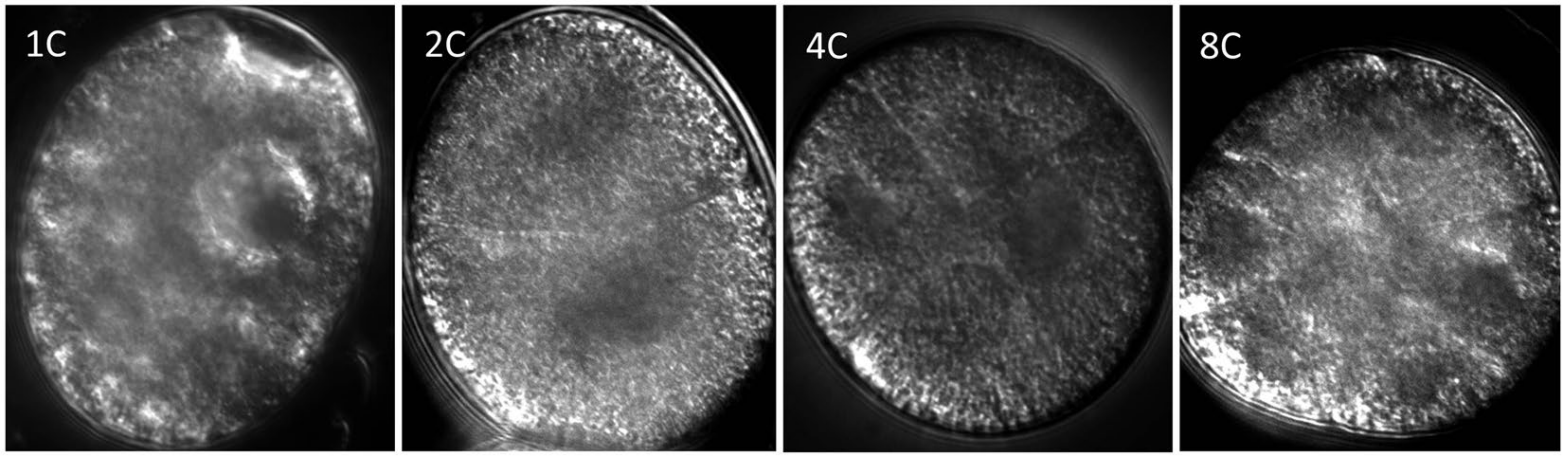
Early cell division (0–4 hours) in *Hypsibius dujardini* embryos maintained at 22 °C, showing nuclear migrations across the one (1 C), two (2 C), four (4 C) and eight (8 C) cell stages.

**Figure 2:**
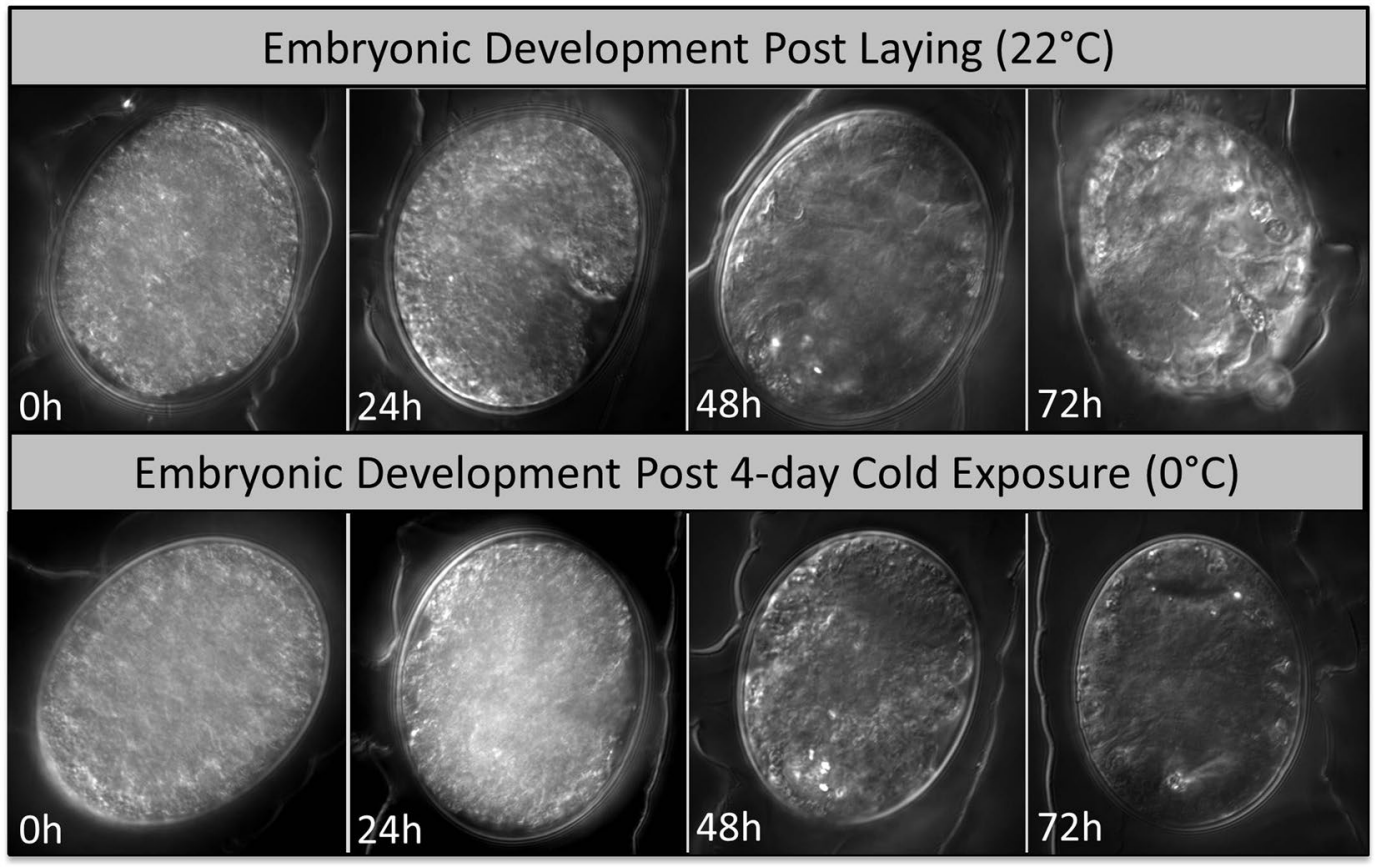
Embryonic development in *Hypsibius dujardini* eggs at 24-hour intervals post laying (22 °C) and post 4-day cold exposure (0 °C).

Cell division in *H. dujardini* embryos is asymmetrical and rapid; nuclear migrations at the 1, 2 4 and 8 cell stages (Fig. 1) occur within the first 4 hours post laying^13^. Embryos typically change from an opaque to a translucent brown color as they develop. This transition in color was observed in all control embryos (22 °C). Control juveniles hatched 4 days post laying and reached sexual maturity approximately 6 days post hatching (N = 30, three trials) – stereotypical development for *H*. *dujardini*. Treatment embryos (0 °C), however, exhibited no color change during the 4-day cold exposure. Once returned to 22 °C, the stereotypical change in color was observed and juveniles hatched 3 days later (Fig. 2). The average egg-to-hatchling period for *H. dujardini* is approximately 4 days^13^; cold-exposed embryos, however, remained at the egg stage for 7 days (4 days at 0 °C + 3 days at 22 °C) before hatching. Thus, while the chronological age for control and cold-treated eggs was equivalent, the biological age for cold-treated eggs was less (i.e., cold treated eggs appeared younger than their control counterparts).

This finding prompted us to consider three hypotheses, potentially with associated lifespan-altering modes, for subsequent testing: eggs exposed to cold then returned to ambient temperature had either (i) retarded, (ii) ceased or (iii) accelerated development. In the first case, (i) exposure to 0 °C had reduced cell division rate – in the extreme, 4 days at 0 °C had been equivalent to one day at room temperature. In the second case, (ii) exposure to 0 °C had induced a developmental checkpoint pathway – in the extreme, cell division halted upon exposure to 0 °C, whereupon a genuine suspended animation ensued. In the third case, (iii) re-introduction to 22 °C had increased cell division rate – in the extreme, 3 days at 22 °C after maintenance in suspended animation at 0 °C for 4 days were equivalent to 4 days at 22 °C. These modes would enable eggs and embryos to tolerate low temperatures for extended time periods and resume cell division and embryogenesis once conditions suitable for development and subsequent growth and reproduction had resumed.

**Figure 3:**
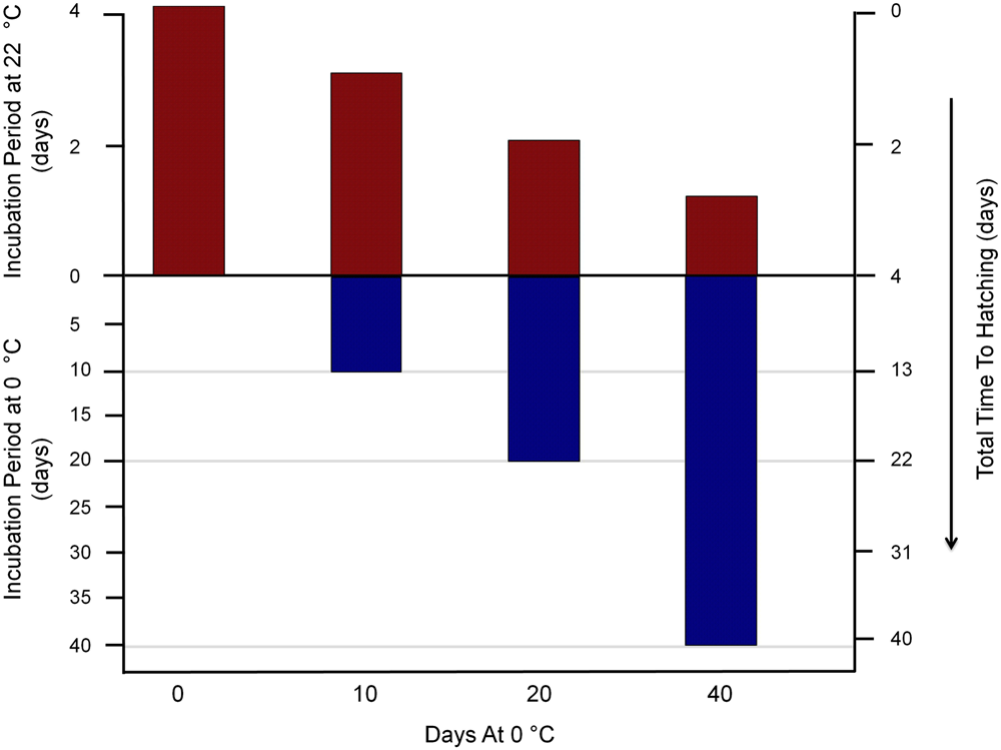
Cold exposure times (x-axis; Days At 0 °C), incubation periods (left y-axis; days) and Total Time To Hatching (right y-axis; days) for *Hypsibius dujardini* eggs (N = 12) exposed to 0 °C for 0, 10, 20 and 40 days. Cold exposure times are interpreted as duration thresholds, incubation periods include exposure to 0 °C (below x-axis) and 22 °C (above x-axis) and Total Time To Hatching includes embryogenesis times at 22 °C (above x-axis) and 0 °C (below x-axis).

As a first step toward distinguishing among the hypotheses and their associated modes, we incubated eggs at 0 °C for longer durations. We found that the lag in development decreased in a quantum-like manner: eggs subjected to 0 °C for a 10-day period and then returned to 22 °C were characterized by a 13-day total incubation period (i.e., at 0 °C and 22 °C; blue and red bar heights relative to left ordinate or ‘y-axis’ in Fig. 3); eggs subjected to 0 °C for a 20-day period and then returned to 22 °C were characterized by a 22-day incubation period (i.e., blue and red bar heights relative to left y-axis in Fig. 3); eggs subjected to 0 °C for a 40-day period were characterized by a 41-day incubation period (i.e., blue and red bar heights relative to left y-axis in Fig. 3). Thus, as cold exposure time (in days) increased, starting at 0 days and doubling from 10 to 20 to 40 (heights for blue bars represented along abscissa or ‘x-axis’ in Fig. 3), incubation periods (in days) at ambient laboratory temperature diminished, starting at 4 and diminishing incrementally from 3 to 2 to 1 (red bar heights relative to left ordinate or ‘y-axis’ in Fig. 3). The most parsimonious interpretation for results obtained with short- and long-term exposure to 0 °C is that *H*. *dujardini* embryos involve a threshold-determined, quantum-like system to control aging. The thresholds occur at exposure durations 0–9, 10–19, 20–39 and ≥40 days (categories along x-axis in Fig. 3). Across the thresholds, embryogenesis time decreases in a quantized manner (red bar heights relative to scale along right y-axis in Fig. 3). Each threshold (i.e., category in Fig. 3) x total incubation period (red + blue bar height in Fig. 3) combination involves equivalent ‘action’ (i.e., energy x time) units.

Lifespan extension resulting from desiccation is most effective as a gradual process, as slow drying enables organisms like rotifers, nematodes and tardigrades to synthesize necessary protectant molecules^1^. Similar to anhydrobiosis, cryobiosis is most effective as a gradual process, providing time for organisms to up-regulate protectant molecules^14^. We found contrastingly that, early in developmental life history, tardigrade embryos respond to cold exposure for different durations by entering a cryobiotic-like state without gradual cooling. This suggests that the observed retardation is a threshold-determined, in addition to quantum-like, action-dependent incremental developmental response (Fig. 3). Tardigrades thus tolerate different extreme conditions through different modes at different stages in life history. Testing whether delayed development leads to lifespan extension remains for future analysis.

We found support for (i) the partial aging hypothesis: development following exposure to 0 °C and return to 22 °C continues but in a diminished manner in the tardigrade species *H. dujardini*; embryos are neither Sleeping Beauties nor Rip van Winkles. To distinguish the cryobiotic responses reported herein from anhydrobiotic responses (and cryobiotic responses to extreme-cold exposures) in adults reported previously, we propose the ‘Snow White’ model: development is retarded in chilled organisms as a consequence from a quantized decrease in average cell division rate.

On the basis of the same incubation period observed for 4- and 10-day cold exposure treatments, we cannot falsify hypothesis (ii); a suspended animation might have been established within each threshold exposure duration (possibly demonstrating an embryonic response in tardigrades similar to Dauer larvae in nematodes^15^). We additionally (and independently) cannot falsify hypothesis (iii) embryogenesis rate might have increased after return to ambient temperature (following cold exposure). All three non-mutually-exclusive hypotheses remain for future, definitive testing.

The processes involved in the cryobiotic responses reported herein might provide means to develop techniques for innovative practical application in cryopreserving eggs from other organisms, most-obviously for human *in vitro* fertilisation. Elucidating the associated mechanisms constitutes another exciting challenge for future research.

## Materials and Methods

To examine effects from exposure to low temperature on tardigrade embryonic development, 20 eggs were collected from 15 egg-laying adults in the parthenogenetic eutardigrade species *Hypsibius dujardini* (Sciento Z151). Eggs were stored in 1.5 mL polypropylene microtubes containing 10 µL spring water and maintained at either 22 °C (control, N = 10) or 0 °C (treatment, N = 10) in a temperature controlled incubator for 4 days (average gestational period). Eggs and embryos maintained at 22 °C were observed daily with light microscopy (Nikon SMZ1000) to assess normal embryonic development. Hatchlings were stored individually in 1.5 mL polypropylene microtubes containing 50 µL spring water and 15 µL algal food (*Chlorococcum* sp.; Sciento A68) and monitored every three days with light microscopy until all had reached sexual maturity (i.e., first embryogenesis). Three trials were conducted. To test long-term cold exposure on embryonic development, additional eggs were maintained either at 22 °C (control, N = 12) or in a temperature-controlled incubator set at 0 °C for 10 (treatment, N = 12), 20 (treatment, N = 12) and 40 days (treatment, N = 12).

For live imaging, embryos were mounted with spring water on uncoated glass microscope 24 × 50 mm No.1 slide covers with thickness 0.13 to 0.17 mm (VWR International). To capture early developmental events, images were captured at the one, two, four and eight-cell stages (approximately 0 to 4 hours post laying; Fig. 1). Embryos maintained at 22 °C were imaged at 24, 48, 72 and 96 hours post laying; embryos maintained at 0 °C were imaged at 24, 48 and 72 hours post 4-day cold exposure (Fig. 2). Specimens otherwise were maintained on the slide covers and placed in a dark, humid box at 22 °C. Development was observed via differential interference contrast (DIC) microscopy with a Plan Apochromat 60 × /1.4 objective lens (Nikon Eclipse Ti), equipped with a high-resolution camera (Photometrics CoolSNAP HQ2 Nikon). Images were viewed and captured with the software NIS Elements AR 4.4 (Nikon Instruments).
